# Enhanced Autophagy in GAB1-Deficient Vascular Endothelial Cells Is Responsible for Atherosclerosis Progression

**DOI:** 10.3389/fphys.2020.559396

**Published:** 2021-02-02

**Authors:** Xin Qian, Han Wang, Yuli Wang, Jiaquan Chen, Xiangjiang Guo, Haoyu Deng

**Affiliations:** Department of Vascular Surgery, Renji Hospital, School of Medicine, Shanghai Jiao Tong University, Shanghai, China

**Keywords:** Gab1, autophagy, endothelia cell, atherosclerosis, peripheral artery disease (PAD)

## Abstract

Autophagy is a host machinery that controls cellular health. Dysfunction of autophagy is responsible for the pathogenesis of many human diseases that include atherosclerosis obliterans (ASO). Physiologically, host autophagy removes aging organelles and delays the formation of atherosclerotic plaque. However, in ischemia event, dysregulated autophagy can be induced to trigger autosis, leading to an inevitable cellular death. Grb2-associated binder 1 (GAB1) is a docking/scaffolding adaptor protein that regulates many cell processes including autophagy. Our study first reported that the protein expression of GAB1 significantly decreased in ASO. Mechanically, our results showed that inhibition of Akt (protein kinase B), the upstream of mTOR (mechanistic target of rapamycin), significantly enhanced autophagy by demonstrating the downregulation of p62/Sequestosome 1 expression and the upregulation of the ratio of LC3II/LC3I. Conversely, we found that the inhibition of ERK1/2 (extracellular signal-regulated kinases1/2), p38, and JNK (c-Jun N-terminal kinase) signaling pathway, respectively, significantly inhibited autophagy by demonstrating the upregulation of p62 expression and the downregulation of the ratio of LC3II/LC3I. Further, we demonstrated that knockdown of GAB1 significantly increased autophagy in HUVECs (human umbilical vein endothelial cells) *via* activation of MAPK (mitogen-activated protein kinase) pathways that include ERK1/2, p38, and JNK. Moreover, we found that knockdown of GAB1 profoundly inhibited HUVEC proliferation, migration, and tube formation. Taken together, this study first suggests that GAB1 is a key regulator of autophagy in HUVECs. Targeting GAB1 may serve as a potential strategy for the atherosclerosis treatment.

## Introduction

Atherosclerosis obliterans (ASO) is a common disease resulting from the occlusion of iliac and femoral arteries. Despite the availability of a plethora of therapies to stabilize atherosclerotic plaque and to recanalize occluded arteries, ASO remains a leading cause contributing to the high morbidity and mortality rate in the elders worldwide (Berger, 2018). The underlying pathogenesis of ASO is widely accepted by the development of atherosclerosis in the peripheral arteries. Pathologically, it is characterized by lipid deposition, endothelial dysfunction, and vascular inflammation (Charo and Taub, 2011). The dysfunction of endothelial cells by lipid deposition is always the earliest event to initiate the process of atherosclerosis (Huang et al., 2019). Generally, it is believed that endothelial cell atherosclerosis is regulated by multiple intracellular signaling pathways that remain to be explored to identify specific targets for drug development. Therefore, our study aim to discover the mechanisms by which intracellular signaling pathways regulate atherosclerosis.

Autophagy is a host machinery that controls cellular health. However, dysfunction of autophagy has been reported to be responsible for many human diseases that include ASO (Evans et al., 2018; Zhou et al., 2018). Autophagy is widely considered as a cellular defense machinery to remove damaged proteins/organelles, maintaining cells alive, but excessive autophagy can result in its own set of problems, such that the cell dies (Grootaert et al., 2018b). It is indicated that autophagy plays an important role in preventing atherosclerosis at the early stage of ASO (Guo et al., 2017; Yao and Qin, 2017; Grootaert et al., 2018a). Moreover, it is reported that autophagy may reduce lipid accumulation and inhibit the formation of foam cells *via* removal of damaged organelles, delaying the development of the plaque (Liao et al., 2012; Razani et al., 2012). Upon atherosclerosis progression, dysregulated autophagy is induced by certain types of ischemia, which further triggers autosis, leading to an inevitable cellular death (Le Guezennec et al., 2012; Maiuri et al., 2013). Taken together, these paradoxical findings questioned us how host machinery determines the specificity and magnitude of autophagy and whether the autophagy is manipulated by diverse host signaling pathways under specific stress.

So far, our knowledge about the signaling regulation of autophagy is relatively limited. Only a few pathways that include mTOR (mechanistic target of rapamycin), AMPK (AMP-activated protein kinase), cAMP-dependent PKA (protein kinase A), and GSK3β (glycogen synthase kinase 3 beta) have been confirmed to be involved in this regulation machinery (Deretic and Klionsky, 2018). Grb2-associated binder 1 (GAB1) is a docking/scaffolding adaptor protein, belonging to the family of insulin receptor substrate 1–like multi-substrate proteins (Wang et al., 2015). Emerging evidences have suggested that signaling pathways mediated by GAB1 play a critical role in the regulation of a variety of cellular processes, including cell proliferation, cell differentiation, apoptosis, and stress responses (Hibi and Hirano, 2000). Disruption of GAB1-mediated signaling pathways has been associated with multiple human diseases, including tumor, cardiovascular disease, and inflammation (Dixit et al., 2005; Shioyama et al., 2011; Zhang et al., 2016). Physiologically, GAB1 functions as a platform for assembling multiple intracellular signaling pathways evoked by various extracellular stimuli *via* its multiple functional domains. Upon activation, GAB1 translocates from cytoplasm to the cellular membrane, where it promotes signaling amplification and transduction by tyrosine phosphorylation and recruitment of downstream proteins including mTOR, AMPK, cAMP-dependent PKA, and GSK3β (Bongartz et al., 2017). Given the importance of GAB1 in regulating autophagic signaling proteins, we questioned that how GAB1 is involved in the pathogenesis of atherosclerosis during ischemic stress.

In this study, we demonstrated that GAB1 is a key regulator of host autophagy. Knockdown of GAB1 significantly increases autophagy in HUVECs (human umbilical vein endothelial cells) *via* MAPK pathways that includes ERK1/2, p38, and JNK. Functionally, we found that knockdown of GAB1 profoundly inhibits HUVEC proliferation, migration, and tube formation. Taken together, our study first suggested that aberrant autophagy induced by loss of GAB1 is responsible for atherosclerosis at a late phase of the disease.

## Materials and Methods

### Patients and Sample Collections

Popliteal arteries from ASO patients who underwent trans-femoral amputation were collected at the Department of Vascular Surgery, Renji Hospital, School of Medicine, Shanghai Jiao Tong University and stored in Biobank of Renji Hospital. All the procedures were approved by the ethics committee of Renji Hospital. Informed consents were obtained before surgical procedures.

### Cell Culture and Lentiviral Particle Transduction

Human umbilical vein endothelial cells were purchased from American Type Culture Collection (PCS-100-010; ATCC, Manassas, VA, United States) and cultured in ECM (#1,001; ScienceCell, United States) supplemented with 5% FBS, 50 ng/ml vascular growth factor, and penicillin/streptomycin (1:1,000). Lentiviral particles were purchased from Genomeditech company in China. The shRNA non-targeting pGMLV SC5 vector with puro resistance and GFP was used as a negative control. For lentiviral infection, HUVECs were incubated with shGAB1 or non-targeting control (2.5 × 10^7^ TU/ml) in ECM supplemented with 10 μg/ml of Polybrene for 48 h. Cells were sorted by puro for 4–5 days followed by further analysis.

### H&E Staining

The tissues were harvested and fixed in 10% formalin, followed by embedding in paraffin and sectioning for standard H&E staining. Each sample was examined by two independent pathologists at five randomized high-magnification fields. The thickness of popliteal artery intima was measured by Image J.

### Immunostaining

Popliteal arteries were fixed with 4% paraformaldehyde for 24 h at room temperature, followed by procession by a serial alcohol gradient and embedment in paraffin wax blocks. The slides were incubated with primary antibodies that include GAB1 Rabbit mAb (#3232; Cell Signaling), LC3B (E5Q2K) Mouse mAb (#83506; Cell Signaling), and Human CD31/PECAM-1 Antibody Sheep mAb (#AF806-SP; R&D) overnight at 4°C, followed by the incubation of secondary antibodies that include donkey anti-rabbit IgG Alexa 488 (#A32790, Invitrogen, green), donkey anti-mouse IgG Alexa plus 647 (#A32787, Invitrogen, pink), and donkey anti-Sheep IgG Alexa 555 (#A-21436, Invitrogen, red). Nuclei were stained with DAPI (#D1306; Invitrogen, Paisley, United Kingdom). An epifluorescence microscope (#DM4000 M; Leica, Wetzlar, Germany) was used for image capture and analysis.

### Cell Proliferation

CCK8 kit (#HY-K0301-500T; MCE, NJ, United States) was used to assess cell proliferation. In brief, cells were seeded in 96-well plates with 5,000 cells per well. CCK8 reagent was added into each well at 0 day, 24 h, 48 h, or 72 h *post*-experiment, respectively, followed by 2-h incubation. The OD value is measured by cell growth curves (#1681135; BioRad, United States) that were mapped with time point as the horizontal ordinate and OD as the longitudinal ordinate.

### Cell Migration Assay

The trans-well system (#3470; Corning, Tewksbury, MA, United States) was employed for cell migration assays as described by the manufacturer. HUVECs (1 × 10^4^) in ECM without FBS or vascular endothelial growth factor were seeded in the upper chambers. The lower chambers were filled with complete ECM. After incubation at 37°C for 24 h, the cells on the upper surface were removed with a cotton swab, while the cells on the undersides of the membranes were stained with gentian violet. The number of the cells was counted under a light microscope in five random visual fields (×200).

### Tube Formation Assay

Tube formation assay was performed as described by the manufacturer. Collagen gels were formed by adding Biocoat-Matrigel (#356234;, Becton Dickinson, Franklin Lakes, NJ, United States) into 96-well plates at 37°C for 30 min. The gels were then overlaid with 2 × 10^4^ cells suspended in culture medium and incubated at 37°C in an atmosphere of 5% CO_2_. Gels were examined by using a phase-contrast microscope equipped with a digital camera PDMC (Polaroid, Minnetonka, MN, United States). Images were captured for the quantification of the number of branches and tubule lengths (defined as those exceeding 200 μm in length) in five randomly chosen fields.

### Western Blot

Cells were harvested using modified oncogene science lysis buffer (250 mM NaCl, 50 mM Tris–HCl, 0.1% Non-idet P-40, 2 mM EDTA, and 10% glycerol) supplemented with protease inhibitors and phosphatase inhibitors. Western blot analysis was performed as previously described (Deng et al., 2017). In brief, equal amounts of proteins were resolved by SDS-PAGE and then transferred to 0.45-μm PVDF membranes. The resulting membranes were incubated with primary antibodies at 4°C overnight, followed by incubation with HRP-conjugated secondary antibodies at room temperature for 1 h. The immunoreactive bands were visualized by enhanced chemiluminescence. Primary antibodies used in this study were as follows: anti-phospho-ERK1/2 (#4370; Cell Signaling), anti-GAB1 (#3232; Cell Signaling), anti-P62 (#16177; Cell Signaling), anti-LC3B (#3868; Cell Signaling), anti-phospho-AKT (#4060; Cell Signaling), anti-phospho-JNK (#9255; Cell Signaling), anti-phospho-HSP27 (#sc-81498; Santa Cruz), and anti-GAPDH (#22549-1-AP; ProteinTech).

### Survival Analysis

The clinical information of 17 patients who underwent trans-femoral amputation were collected. The patients were followed up *via* telephone to record their clinical outcome. The survival analysis was determined by Kaplan–Meier analysis and plotted by GraphASO 7.0.

### Statistical Analysis

All results presented are representative of at least three independent experiments. Results generated from *in vitro* experiments are expressed as mean ± SDs. Statistical analysis was conducted using unpaired Student’s *t* test. Values of *p* < 0.05 were considered to be statistically significant.

## Results

### Loss of GAB1 Is Associated With Atherosclerosis in ASO

**FIGURE 1:**
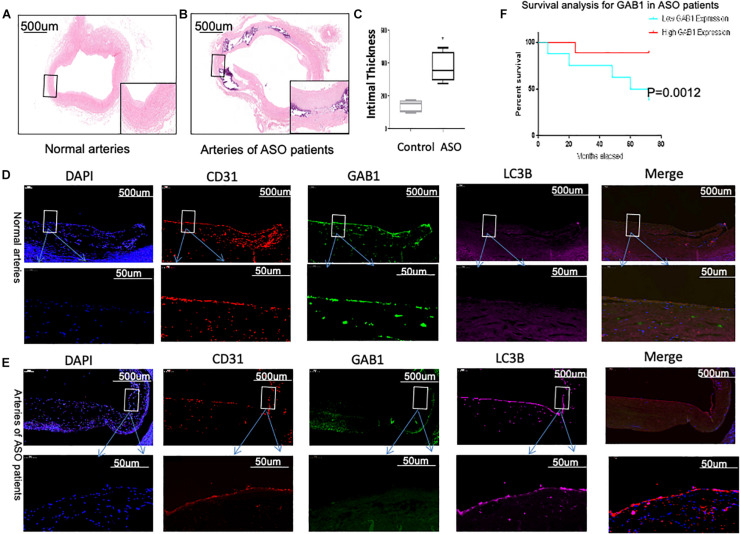
GAB1 decreased in vascular endothelia and is closely associated with patients’ survival. **(A,B)** H&E staining of normal artery and ASO artery. **(C)** H&E staining showed the thickness of intima increased in ASO artery. **(D,E)** GAB1 decreased in endothelia of ASO, which was identified by immunostaining of normal artery and ASO artery. Endothelial cells were identified by CD31 (red), GAB1 (green), and LC3B (pink) that were counter-stained with DAPI (blue). **(F)** Survival analysis for GAB1 was made and showed low GAB1 expression predicted poor prognosis in ASO patients. **(D,E)** Results are presented as mean ± SD, ^∗^*p* < 0.05.

We first determined the intima thickness in both ASO and control groups by H&E staining. The results demonstrated that intima thickness in ASO group significantly increased as compared with control groups (Figures 1A–C). We then questioned whether the protein expression of GAB1 is different between two groups. Confocal microscopy analysis demonstrated that GAB1 expression profoundly decreased in the intima of popliteal arteries, together with an increased expression of LC3II in ASO patients as compared with healthy controls (Figures 1D,E). Given the importance of GAB1 in the development of atherosclerosis, we further performed survival analysis to determine whether GAB1 expression is responsible for long-term survival of ASO patients. Kaplan–Meier results showed that ASO patients with a high expression level of GAB1 obtained a significant survival benefit as compared with those with a low expression level of GAB1. Taken together, these results suggested that loss of GAB1 is associated with atherosclerosis in ASO.

### GAB1 Negatively Regulates Autophagy of Vascular Endothelial Cells

**FIGURE 2:**
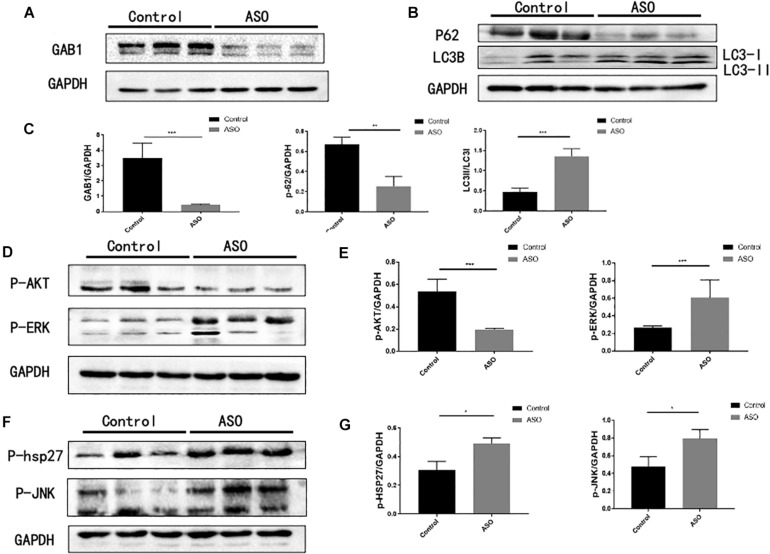
The expression of GAB1 and autophagy-mediated pathways in ASO artery. **(A)** GAB1 was decreased in ASO artery by Western blot assay. **(B)** Autophagy was enhanced in ASO artery by Western blot assay with markers of P62 and the ratio of LC3II/LC3I. **(C)** Statistical analysis of GAB1, P62, and LC3II/LC3I. **(D)** P-AKT was decreased but P-JNK was increased in ASO artery by Western blot assay. **(E)** Statistical analysis of P-AKT and P-JNK. **(F)** P-HSP27 and P-ERK was increased in ASO artery by Western blot assay. **(G)** Statistical analysis of P-HSP27 and P-ERK. Results are presented as mean ± SD, ^∗^*p* < 0.05, ^∗∗∗^*p* < 0.001.

Further, Western blot analysis confirmed that the protein expression of GAB1 in intima of ASO popliteal arteries significantly decreased as compared with that of controls, suggesting that translation of GAB1 is destroyed upon atherosclerosis (Figure 2A). We next determined autophagic activity of vascular endothelial cells between ASO and healthy control groups. In this study, we chose p62 degradation and LC3-phosphatidylethanolamine complex, commonly referring to LC3-II as two markers of autophagy. SQSTM1/p62 is an autophagy receptor targeting ubiquitinated proteins to autophagosomes for degradation (Bongartz et al., 2017), while LC3-II participates in the formation of autophagosomes and remains attached to matured autophagosomes. Figures 2B,C demonstrated that protein level of p62 significantly decreased in the ASO group as compared with the control group. Conversely, the ratio of LC3-II/LC3-I significantly increased in ASO group as compared with control group (Figures 2B,C). These results together suggest that autophagic activity of vascular endothelial cells is activated upon atherosclerosis. Further, we evaluated whether GAB1-mediated intracellular signaling is activated during autophagy. Our results found that phosphorylation of ERK1/2, p38, and JNK are significantly increased, while phosphorylation of Akt are significantly decreased in ASO group as compared with control group, indicating that these four intracellular pathways may be involved in the GAB1-mediated autophagy modification (Figures 2D–G).

### Intracellular Signaling Pathways Are Responsible for Autophagy Regulation

**FIGURE 3:**
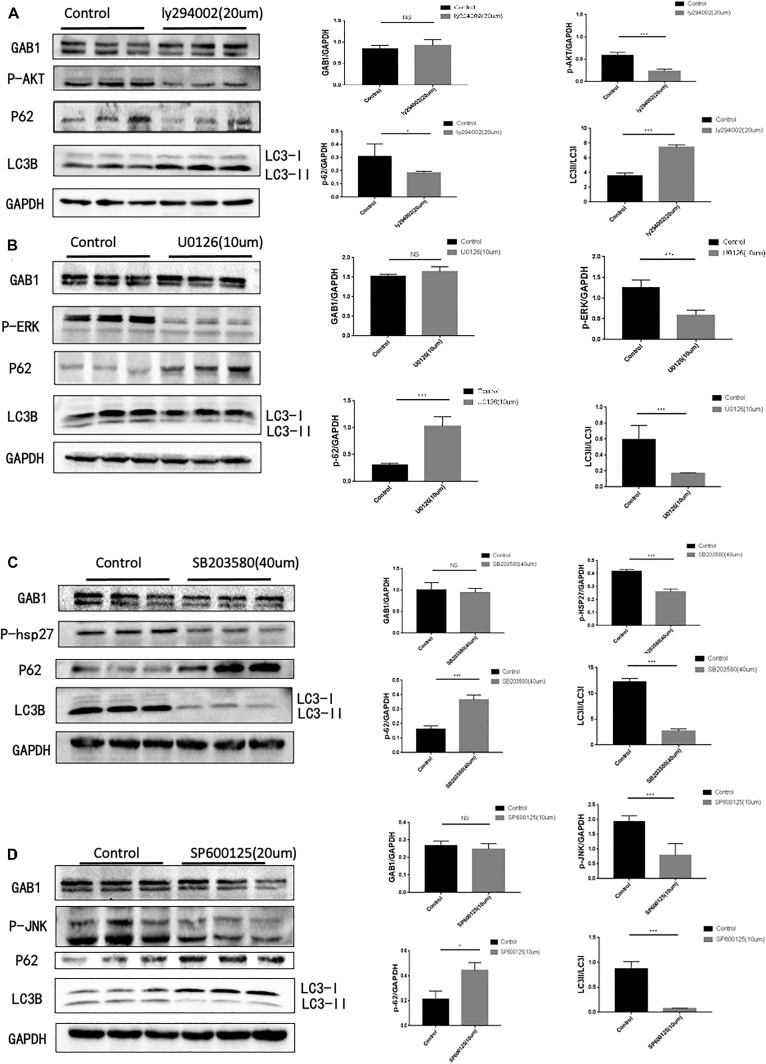
GAB1 may act as upstream of PI3K/AKT, P38, JNK, and ERK, which are key pathways in autophagy induction. **(A)** Autophagy was inhibited with markers of elevated ratio of LC3II/LC3I and decreased P62 while GAB1 did not change when 20 μm PI3K inhibitor was added into medium and the statistical analysis was made. **(B)** Autophagy was enhanced with markers of decreased ratio of LC3II/LC3I and elevated P62 while GAB1 did not change when 10 μm ERK inhibitor was added into medium and the statistical analysis was made. **(C)** Autophagy was enhanced with markers of decreased ratio of LC3II/LC3I and elevated P62 while GAB1 did not change when 40 μm P38 inhibitor was added into medium and the statistical analysis was made. **(D)** Autophagy was enhanced with markers of decreased ratio of LC3II/LC3I and elevated P62 while GAB1 did not change when 20 μm JNK inhibitor was added into medium and the statistical analysis was made. Results are presented as mean ± SD, ^∗^*p* < 0.05, ^∗∗∗^*p* < 0.001.

As GAB1 works as a hub for intracellular signaling assembly and transduction, we next questioned how GAB1 regulates its downstream signaling molecules for autophagy regulation (Figure 3). We performed the experiments using different signaling inhibitors that include PI3K inhibitor, ly294002, ERK1/2 inhibitor, U0216, p38 inhibitor, SB203580, and JNK inhibitor, and SP600215 to study the functional role of each signaling pathway. It is well established that activation of mTORC1 by nutrients and growth factors leads to inhibition of autophagy. Similarly, our results showed that inhibition of Akt, the upstream of mTOR, significantly enhanced autophagy by demonstrating the downregulation of p62 protein expression and the upregulation of the ratio of LC3II/LC3I. Conversely, we demonstrated that inhibition of ERK1/2, p38, and JNK significantly destroyed autophagy by demonstrating the upregulation of p62 protein expression and the downregulation of the ratio of LC3II/LC3I.

### Knockdown GAB1 Promotes Autophagy in HUVECs *via* Upregulation of p-ERK, p-p38, and p-JNK

**FIGURE 4:**
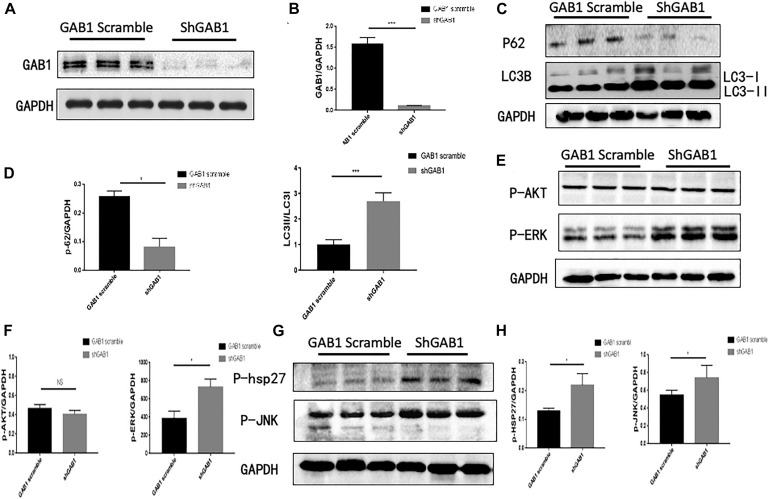
GAB1 knockdown increased autophagy induction and the activity of P38, JNK, and ERK. **(A,B)** The expression of GAB1 after being transfected lentivirus and its statistical analysis. **(C,D)** The expression of P62 and LC3B after being transfected GAB1 knockdown lentivirus and its statistical analysis. **(E,F)** The expression of P-AKT and P-ERK after being transfected GAB1 knockdown lentivirus and its statistical analysis. **(G,H)** The expression of P-JNK and P38 after being transfected GAB1 knockdown lentivirus and its statistical analysis. Results are presented as mean ± SD, ^∗^*p* < 0.05, ^∗∗∗^*p* < 0.001.

Considering the fact that ERK1/2, p38, and JNK signaling are involved in the regulation of autophagy in HUVECs, we questioned if GAB1 plays a dominant role in determining cell fate by autophagy process. We first used a lentivirus expressing siGAB1 to knock down endogenous GAB1 in HUVECs. The Western blot results showed that shGAB1 transfection significantly decreased the protein expression of GAB1 in HUVECs, indicating a successful procedure (Figures 4A,B). Next, we determined the protein expression of LC3I/II and p62 using Western blot. Figures 4C,D demonstrated that knockdown of GAB1 significantly decreased the protein expression of p62, while it increased the ratio of LC3II/LC3I. These results indicated that knockdown of GAB1 promotes autophagy in HUVECs. On the other hand, we also determined whether knockdown of GAB1 has an impact on its downstream signaling. The results demonstrated that knockdown of GAB1 significantly upregulates the phosphorylation level of p-ERK1/2, p-p38, and p-JNK. However, little significance was observed on the phosphorylation level of p-Akt. These results are in accord with the experiments using signaling inhibitors, suggesting that GAB1 may regulate autophagy *via* intracellular signaling of p-ERK, p-p38, and p-JNK (Figures 4E–H).

### GAB1 Promotes HUVEC Proliferation and Migration

**FIGURE 5:**
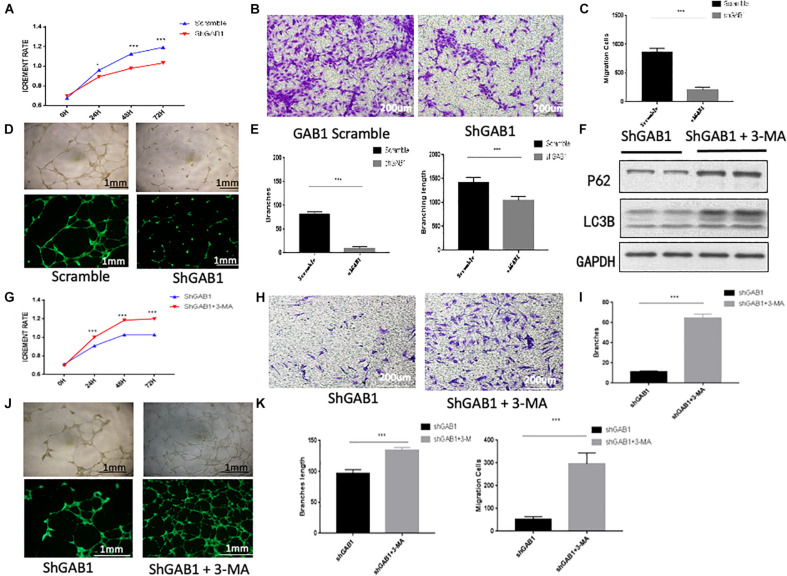
The influence of GAB1 on endothelial function. **(A)** CCK8 assay showed GAB1 knockdown impaired endothelial proliferation. **(B,C)** GAB1 knockdown damaged endothelial migration significantly. **(D,E)** GAB1 knockdown also injured endothelial tube formation including branches and branching length. **(F)** The expression of P62 and LC3B in shGAB1 HUVECs with or without 3-MA in medium. **(G)** GAB1 knock down increased endothelial proliferation with 3-MA in medium. **(H,I)** GAB1 knockdown promoted endothelial migration significantly with 3-MA in medium. **(J,K)** GAB1 knockdown also upregulated endothelial tube formation including branches and branching length with 3-MA in medium and the statistical analysis was made. Results are presented as mean ± SD, ^∗^*p* < 0.05, ^∗∗∗^*p* < 0.001.

Previous work using transmission electron microscopy showed characteristics of autophagy in dying endothelial cells (Lenoir et al., 2015). However, further studies are needed to illustrate whether knockdown of GAB1 impairs endothelial function *via* autophagy. We therefore performed endothelial function experiments in HUVECs using GAB1 knockdown lentivirus. The results showed that knockdown of GAB1 significantly decreased endothelial function including cell proliferation, migration, and tube formation (Figures 5A–E). Moreover, shGAB1 HUVECs were treated with PBS or autophagy inhibitor 3-MA (5 mM),respectively, to perform rescue experiments. As compared with 3-MA group, autophagy was significantly enhanced in shGAB1 HUVECs by demonstration of an elevated ratio of LC3II/LC3I and a reduced protein expression of p62 (Figure 5F). We also observed that HUVEC proliferation was significantly upregulated in the 3-MA-treated group as compared with the control group, suggesting that GAB1 plays an important role in maintaining cell survival *via* autophagy (Figure 5G). Further, we performed cell migration assay, the results showed that knockdown of GAB1 significantly inhibited cell migration rates (Figures 5H,I). In addition, we found that tube formation rate of HUVECs was significantly decreased in shGAB1 group, as compared with shGAB1 + 3-MA treated group, indicating that GAB1 is a key regulator in vasculature maintenance, and autophagy may be the key regulatory mechanism (Figures 5J,K).

## Discussion

Grb2-associated binder 1, as an adaptor protein, has several domains within its structure that allow it to interact with other proteins such as insulin receptor and fibroblast growth factor receptor substrate two complex (Wang et al., 2015; Xu et al., 2016). After being activated, GAB1 participates in various signaling pathways, including Ras–ERK and PI3K–AKT pathways (Rajadurai et al., 2012). Due to its special structure, GAB1 has been implicated in several pathophysiological processes of different diseases that include tumorigenesis and metastasis (Mraz et al., 2014; Furuta et al., 2016; Zhang et al., 2016). But in ASO, little studies has showed a positive association between dysfunction of GAB1 and atherosclerosis (Raghavan et al., 2018). Therefore, our study aims to establish the relationship between GAB1 dysfunction and the onset of atherosclerosis. Of interest, our study first reported that downregulation of GAB1 in arteriosclerotic arteries was closely associated with ASO progression and subsequent patients’ survival.

It is well established that both PI3K/Akt and MAPK pathways that include ERK, p38, and JNK signaling are key regulators in autophagy process upon diverse stimulations. PI3K/Akt is a classic signaling that inhibits autophagy by blocking autophagosome formation and plays a critical role in many diseases *via* regulating the level of autophagy, such as melanoma, Parkinson’s disease, and rheumatoid arthritis (Wang et al., 2012; Feng and Qiu, 2018; Liang et al., 2019). ERK is generally believed to play a more important role in inducing autophagic programmed cell death rather than apoptosis in neuron cell death and ovarian cancer cells (Chu et al., 2004; Subramaniam and Unsicker, 2006). JNK, a stress-activated protein kinase, is reported to respond to stress signals to enhance autophagy by promoting the dissociation of the Beclin 1-Bcl-2/Bcl-xL (Sun et al., 2011; Zhao et al., 2011). Interestingly, p38 signaling pathway has both positive and negative effect on autophagy. On the one hand, p38 signaling is responsible for the maintenance of cell survival; on the other hand, it can also negatively regulate basal autophagy *via* the blockage of Atg9 to form autophagosomes (Cui et al., 2007; Ye et al., 2011; Choi et al., 2012). In our study, our results showed that the inhibition of ERK, P38, and JNK pathways significantly destroyed the formation of host autophagy, respectively, while the inhibition of PI3K/Akt pathway can promote the autophagy. To further identify the relationship between GAB1 and autophagy, we performed a GAB1 knockdown experiment by lentivirus in HUVEC. We found that knockdown of GAB1 significantly enhanced autophagy formation. Functionally, we demonstrated that knockdown of GAB1 impaired cell proliferation, cell migration, and tube formation of HUVECs, which is believed to be a potential mechanism contributing to the development of atherosclerosis.

**FIGURE 6:**
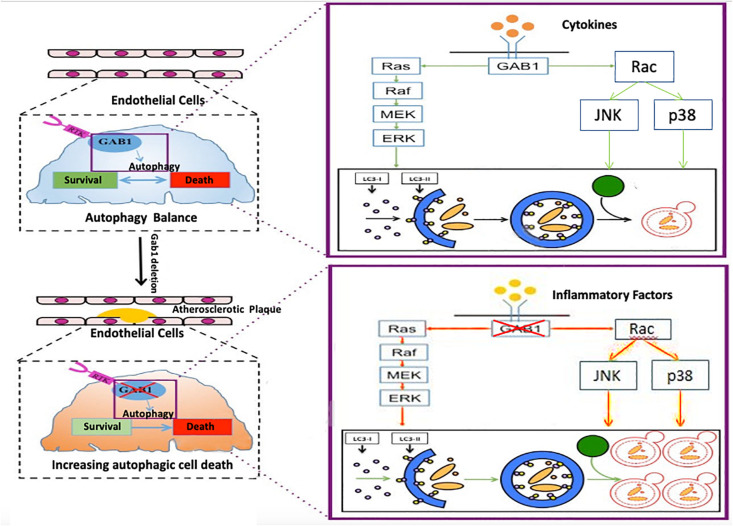
The mechanism by which GAB1 knockdown promotes autophagy in ASO GAB1 regulated autophagy balance to maintain cell survival and function in normal artery. When atherosclerosis occurred, GAB1-mediated autophagy lost balance and excess autophagy led to endothelial dysfunction in response to inflammatory cytokines by increasing the activity of ERK, P38, and JNK pathways, which gave new insight into the mechanisms of atherosclerosis.

Taken together, our study first found that dysfunction of GAB1 is responsible for HUVEC malfunction *via* excessive autophagy activation (Figure 6). This finding complements the current understanding of autophagy in the late stage of atherosclerosis, which shed light on a potential strategy for drug development to treat ASO by targeting GAB1.

## Data Availability Statement

The original contributions presented in the study are included in the article/Supplementary Material, further inquiries can be directed to the corresponding author/s.

## Ethics Statement

The studies involving human participants were reviewed and approved by Renji Hospital, School of Medicine, Shanghai Jiao Tong University. The patients/participants provided their written informed consent to participate in this study.

## Author Contributions

HD designed the studies. XQ, HW, YW, JC, and XG performed the experiments. HD and XQ wrote and revised the manuscript. All authors contributed to the article and approved the submitted version.

## Conflict of Interest

The authors declare that the research was conducted in the absence of any commercial or financial relationships that could be construed as a potential conflict of interest.
