# Supplementary material for: Enhanced Autophagy in GAB1-Deficient Vascular Endothelial Cells Is Responsible for Atherosclerosis Progression
Source: Front Physiol. 2021 Feb 2;11:559396. doi: 10.3389/fphys.2020.559396 (PMC7877249; doi:10.3389/fphys.2020.559396)
Supplement: Supplementary file 1 [file Data_Sheet_1.PDF]

Baseline in patients grouped by relative Gab1 fluorescence intensity

	ALL n=17	Low expression n=9	High expression n=8	p-Value
Age(years)	72[60-85]	75[63-82]	68[60-85]	0.571
Men	17(100%)	9（100%）	8（100%）	
Hypertension	12 (70.6%)	7(77.8%)	5(62.5%)	0.886
Diabetes	7(41.2%)	4(44.4%)	3(37.5%)	0.667
ALT(U/L)	18.93[5.68-32.18]	18.15[7.61-28.69]	19.81[4.76-34.86]	0.943
Cr(umol/L)	77.5[68.17-86.83]	72.3[62.21-82.39]	83.35[43.47-123.23]	0.462
Triglyceride(mmol/L)	1.83[0.72-2.94]	1.97[0.28-4.46]	1.67[0.91-2.43]	0.623
HsCRP(mg/L)	2.68[1.02-4.34]	2.72[0.71-4.73]	2.64[1.12-4.16]	0.781
BNP(pg/ml)	83 [65.17-100.83]	81.96[32-131.92]	84.17[46.25-122.09]	0.644

Supplementary table
